# Recycling of predictors used to estimate glomerular filtration rate: Insight into lateral collinearity

**DOI:** 10.1371/journal.pone.0228842

**Published:** 2020-02-11

**Authors:** Luis Gustavo Modelli de Andrade, Helio Tedesco-Silva

**Affiliations:** 1 Department of Internal Medicine, UNESP, Univ Estadual Paulista, Botucatu, Brazil; 2 Hospital do Rim, Universidade Federal de São Paulo, So Paulo, Brazil; Istituto Di Ricerche Farmacologiche Mario Negri, ITALY

## Abstract

**Background:**

One overlooked problem in statistical analysis is lateral collinearity, a phenomenon that may occur when the outcome variable derives from the predictors. In nephrology this issue is seen with the use of estimated glomerular filtration rate (eGFR) as an outcome and age, sex, and ethnicity as predictors. In this study with simulated data, we aim to illustrate this problem.

**Methods:**

We randomly generated unrelated data to estimate eGFR by common equations.

**Results:**

Using simulated data, we show that age, gender, and ethnicity (recycled predictors variables) are statistically significantly correlated with eGFR in linear regression analysis. Whereas the initial obvious conclusion is that age, sex, and ethnicity are strong predictors of eGFR, more rigorous interpretation suggests that this is a byproduct of the mathematical model produced when deriving new predictors from another.

**Conclusion:**

While statistical models have the ability to identify vertical collinearity (predictor-predictor), lateral collinearity (predictor-outcome) is seldom identified and discussed in statistical analysis. Therefore, caution is needed when interpreting the correlation between age, gender, and ethnicity with eGFR derived from regression analyses.

## Introduction

The estimated glomerular filtration rate (eGFR), calculated using age, ethnicity, gender and creatinine values in the MDRD [1] and CKD-EPI [2] equations, has been used as an appropriate surrogate for glomerular function [3]. The eGFR has been used as an outcome in several epidemiological studies [4–5] including in transplantation [6] and is frequently considered as a surrogate marker in clinical studies.

The use of age, ethnicity, and gender as “new” predictors of an eGFR outcome may introduce a bias because these variables are already used in the mathematical equations. One of the assumptions of the regression analysis is that the predictor variables are independent [7] and that there is no strong collinearity between the predictors [8] (absence of vertical collinearity). On the other hand, Shinoda et al [6] shows that age correlates with eGFR at one year after kidney donation, yet age is used to determine eGFR, leading to a phenomenon called lateral collinearity [8]. Here we discuss the influence of collinearities in the interpretation of eGFR association studies.

## Methods

We simulated baseline data for 1000 patients (age, ethnicity, gender, and creatinine) and calculated eGFR by MDRD [1] and CKD-EPI [2] equations. Simulated data of continuous variables (age and creatinine) were obtained from a normal distribution. For categorical variables, the distribution was empirically chosen in 50% of sex (male/female) and 30% of black ethnicity. Creatinine values below 0.5mg/dl (9% of the sample) were replaced by 0.5 mg/dl.

### Statistics

The correlations between continuous variables were performed with the Pearson’s correlation coefficient. Linear regression models were constructed to evaluate the relationship between predictors (age, sex, and ethnicity) with creatinine and these predictors with eGFR. Collinearity analysis was performed with the variance inflation factor (VIF) of the individual predictor variables, with values below 2 indicating a low degree of collinearity and 10 or higher extreme collinearity [8]. Analyzes were performed using R software version 3.4.2 (S1 File).

## Results

**Fig 1:**
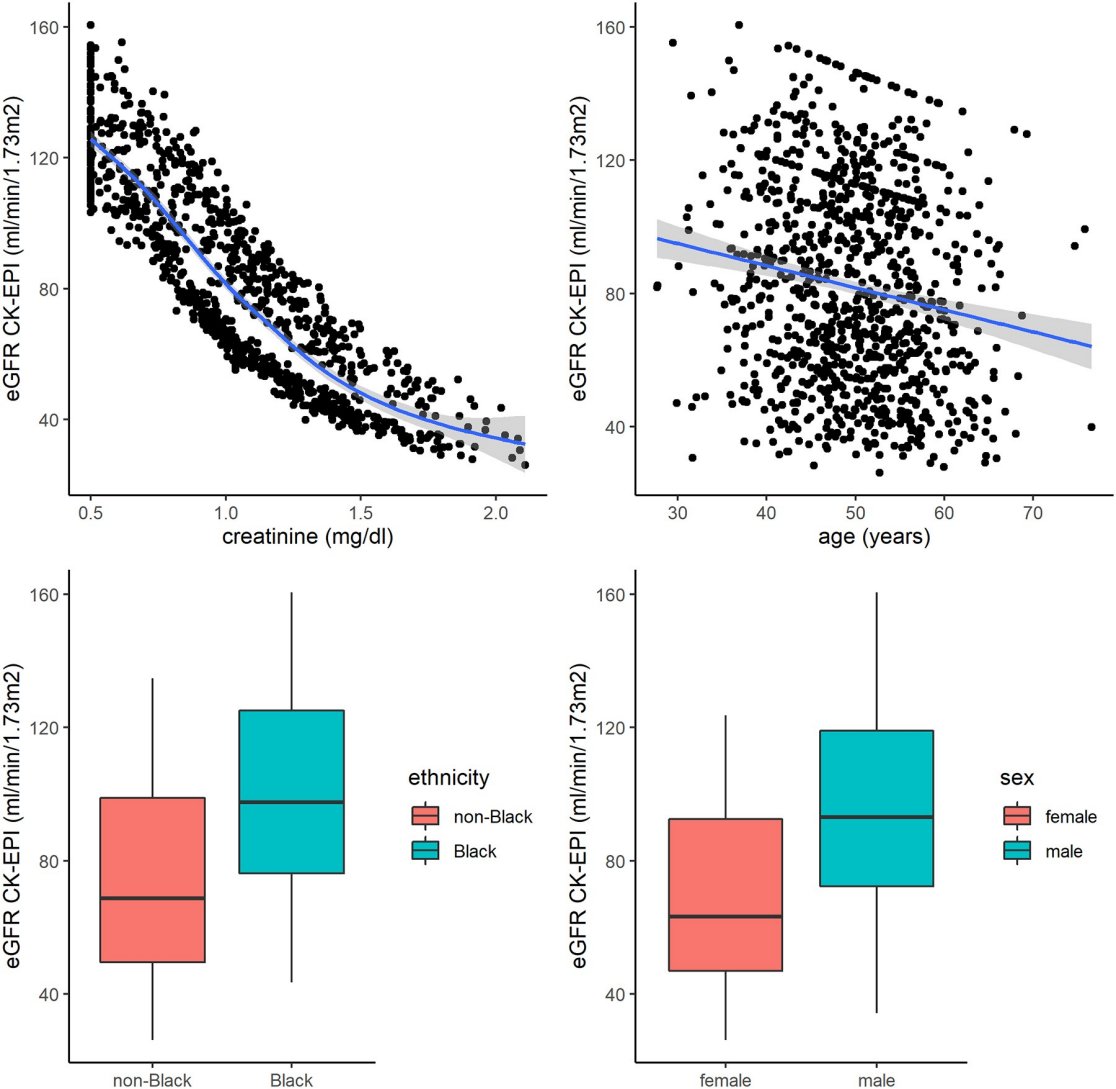
The relationship between the age, creatinine, sex and ethnic with estimated glomerular filtration rate (eGFR). The Pearson coefficients were: creatinine (r = -0.87, p <0.001), age (r = -0.16, p < 0.001), gender male (r = 0.43, p <0.001), and non-black ethnicity (r = -0.38, p <0.001).

The mean age was 50±8 years and the mean creatinine value was 1±0.38 mg/dl (Table 1). There was no prior correlation between the random generated variables. As expected, the predictor variables creatinine (r = -0.87, p <0.001), age (r = -0.16, p < 0.001), non-black ethnicity (r = -0.38, p <0.001), and male gender (r = 0.43, p <0.001) were associated with eGFR using the CKD-EPI (Fig 1).

**Table 1:** Simulated values and estimated glomerular filtration rate in a simulated sample.

	Overall (N = 1000)
**Age (ys)**	
Mean (SD)	49.763 (7.631)
Range	27.680–76.584
**Sex**	
female	500 (50.0%)
male	500 (50.0%)
**Creatinine (mg/dl)**	
Mean (SD)	1.043 (0.363)
Range	0.500–2.109
**ethnicity**	
non-Black	700 (70.0%)
Black	300 (30.0%)
**eGFR CKD-EPI (ml/min/1.73m2)**	
Mean (SD)	81.906 (30.548)
Range	26.128–160.583
**eGFR_MDRD** **(ml/min/1.73m2)**	
Mean (SD)	83.820 (43.298)
Range	24.546–226.915

While there was no correlation of predictor variables (age, gender, and ethnicity) with creatinine, as expected by the random nature of the data, a correlation between these predictors with the eGFR by CKD-EPI and by MDRD was observed after performing a linear regression model (Table 2). When creatinine values are included in this statistical model, all predictor variables were significantly correlated with the eGFR CKD-EPI outcome (Table 3). We also simulated models with the same sample size but with correction factors of 0.7 for females and 1.2 for blacks, derived from the MDRD equation, and a decrease of creatinine values with age as an exponential function (exp -0.2). These sensitive analyses also showed associations between baseline variables with the eGFR CKD-EPI (S2 File), confirming the lateral collinearity.

**Table 2:** Linear model of age, sex and ethnicity with creatinine (outcome) and eGFR by MDRD and CKD-EPI (simulated data).

	creatinine (mg/dl)			eGFR MDRD (ml/min/1.73m^2^)			eGFR CKD-EPI (ml/min/1.73m^2^)		
	*B*	*CI*	*p*	*B*	*CI*	*p*	*B*	*CI*	*p*
(Intercept)	0.97	0.82–1.12	< .001	83.61	67.59–99.64	< .001	103.14	92.06–114.22	< .001
Age (ys)	0.00	-0.00–0.00	.236	-0.37	-0.69 –-0.06	.021	-0.69	-0.91 –-0.47	< .001
Sex (male)	-0.03	-0.09–0.03	.291	27.91	21.56–34.26	< .001	18.79	14.40–23.18	< .001
Ethnicity (Black)	0.00	-0.06–0.07	.912	15.62	8.69–22.55	< .001	12.78	7.98–17.57	< .001
Observations	1000			1000			1000		
R^2^ / adj. R^2^	.003 / .000			.205 / .202			.236 / .234		

**Table 3:** Linear model of age, creatinine, sex and ethnicity with eGFR by CKD-EPI (simulated data). VIF: variance inflation factor.

	eGFR CKD-EPI (ml/min/1.73m^2^)			
	*B*	*CI*	*p*	*VIF*
(Intercept)	172.70	169.97–175.44	< .001	
Age (ys)	-0.56	-0.61 –-0.51	< .001	1.00
sex (male)	16.48	15.48–17.49	< .001	1.75
Creatinine (mg/dl)	-71.76	-72.81 –-70.71	< .001	1.00
ethnicity (Black)	13.04	11.94–14.14	< .001	1.75
Observations	1000			
R^2^ / adj. R^2^	.960 / .960			

The VIF values for all predictor variables were less than 2, suggesting no collinearity (Table 3).

## Discussion

**Fig 2:**
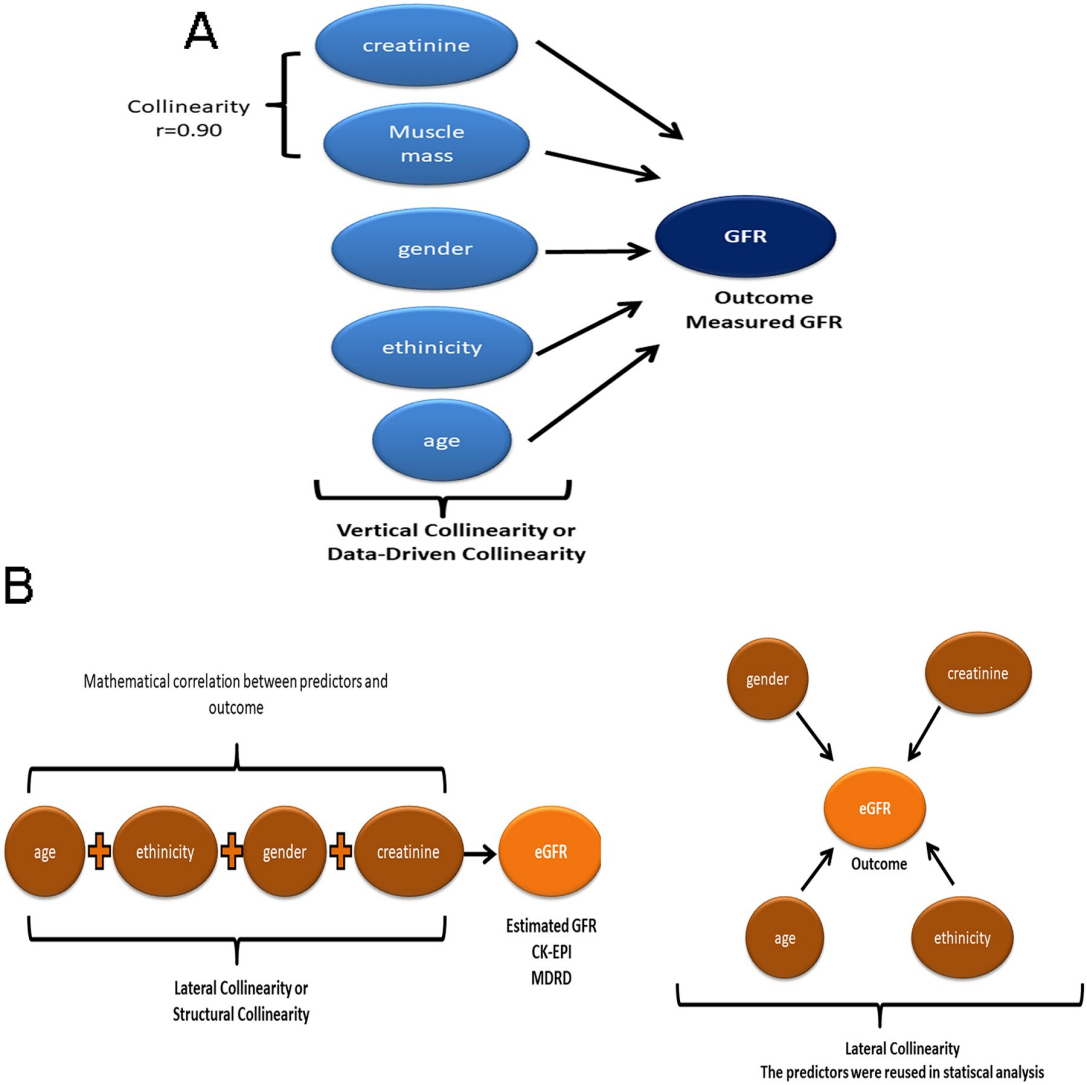
The two types of collinearity in statistical. A: Vertical or data-driven collinearity occurs when the predictors are correlated with others. This can assess by a correlation coefficient. In this example, the muscle mass was correlated with creatinine assess by a Pearson coefficient of 0.9. B: Lateral or structural collinearity occurs when the predictors are mathematically correlated with the outcome and are reused as new predictors. In this example, the creatinine, age, gender, and ethnicity were correlated by a mathematical equation used to estimate GFR. The predictors were reused in a new model with the eGFR as the outcome and we expected an artificial correlation.

Multivariable regression models are subjected to two types of collinearity effects. The vertical collinearity is the “classic” type, referring to predictor-predictor collinearity and can be identified by higher values of VIF [9] (Fig 2A). Because there is an objective way of measure, this type of collinearity is generally avoided in statistical models. On the other hand, the predictor variables may also be collinear with the outcome variable, a phenomenon called lateral collinearity (Fig 2B). This collinearity is caused by a mathematical artifact when a new predictor is derived from one or more predictive variables. Lateral collinearity is rarely explicitly tested in multivariable analyses [9].

This simulation analysis shows that creatinine, age, ethnicity, and gender are not independently associated with eGFR but a result of lateral collinearity (Fig 2). Such misleading associations have been described in several studies (Das et al [10] and Stapleton et al [11]). We also considered that the collinearity arises when the predictors used to estimate GFR were present in the equations. For MDRD and CKD-EPI, the predictors were age, creatinine, sex, and ethnicity, but this could be different for other equations. For example, the full age spectrum equation (FAS) the predictors used were age, creatinine, and sex [12]. Therefore, collinearity will not be present when ethnicity is used to estimate eGFR using this equation. Furthermore, in several populations such as anorectic, cirrhotic, obese, renal and non-renal transplant patients, the eGFR performance is poor, as highlighted by Delanaye et al [13]. Altogether, the use of eGFR to estimate glomerular function as an outcome has two major methodological weaknesses, population-derived bias and the lateral collinearity. To access the true association between age and renal function, the measured GFR by iohexol is simple and can provide an unbiased estimative of GFR [13].

In order to reduced lateral collinearity we must avoid the reuse of predictors that were already used to estimate the outcome. Then this type of bias occurs when the outcome is derived from the predictors [4–6] as in body mass index (BMI), which is calculated as a function of height, and weight, and the KDPI, which is calculated using 6 donors variables. As an example of lateral collinearity in the study by Das et al [10], glomerular filtration was estimated with laboratory data and conclusions are drawn from regression based on age, ethnicity, and sex, variables that are also used to estimate GFR. Similarly, in a recent study by Stapleton et al [11], the eGFR calculated by CKD-EPI was used as the outcome variable and the log_10_eGFR was correlated with several predictors including recipient age [11]. In this example, the explanatory capacity of the statistical model has been compromised. That is, we cannot assume conclusions such as the correlation between increasing recipient age and reducing eGFR because this relationship was artificially created by the equation used to estimate GFR. This is an example of lateral collinearity that should be suspected when we derive the outcome from the predictors. This relationship is not identified when performing traditional collinearity measures such as the VIF.

**Fig 3:**
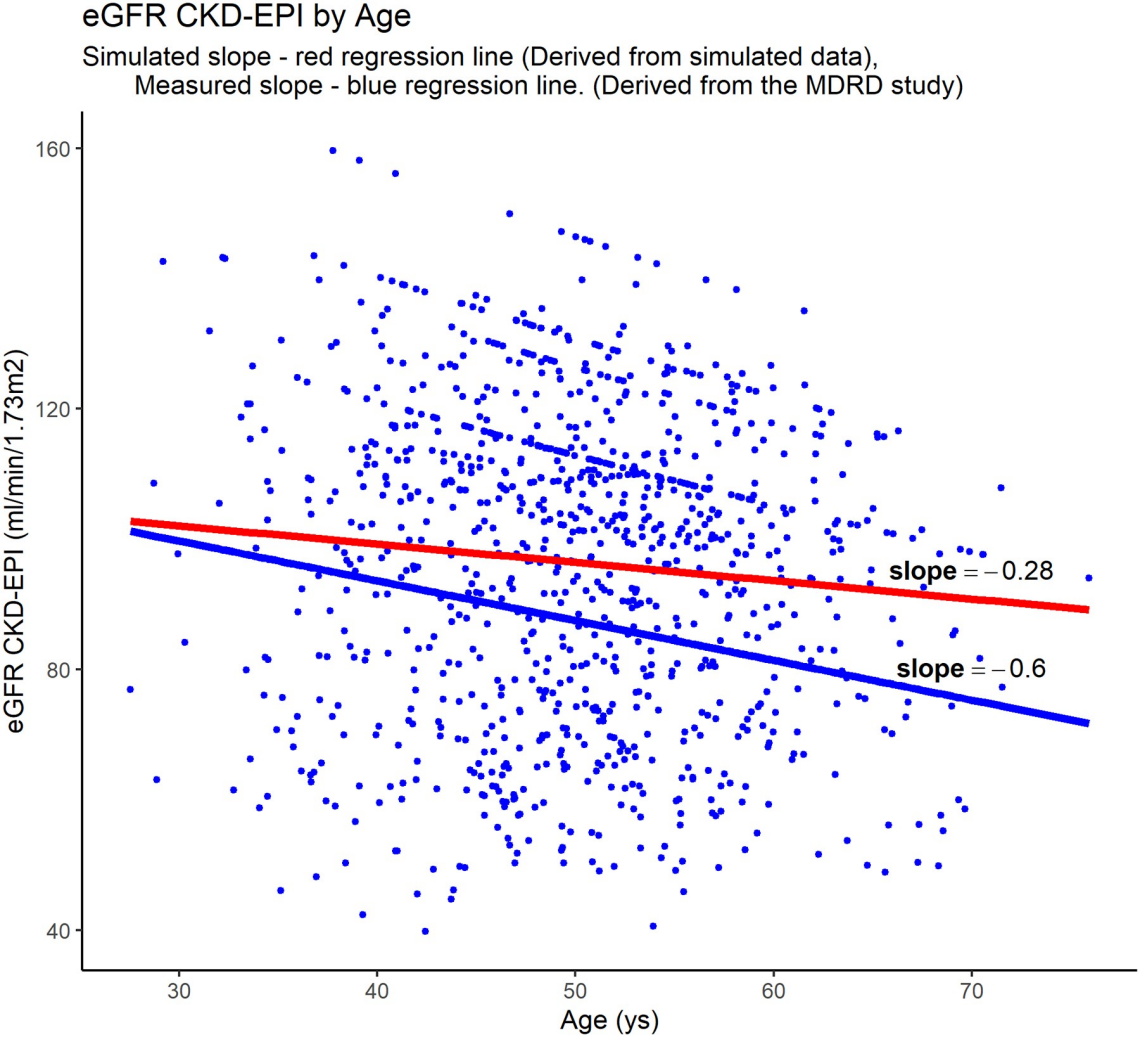
Relationship between eGFR by CKD-EPI with age in a simulated epidemiologic study. The expected decline in renal function was obtained from the MDRD study. In this study, the expected decline in renal function by age follows an exponential function. In the graph, the blue regression line represents the expected slope of renal function decline (-0.6ml / min/1.73m^2^/year). The simulated slope is the red regression line (-0.28 ml/min/1.73m^2^/year) and represents the values obtained with the simulated data. The values obtained with the simulated data underestimating the true decline in renal function.

The most important misleading effect introduced by lateral collinearity occurs when we infer statistical associations between baseline variables and estimated GFR. This may occur in epidemiological studies attempting to associate increasing age with reduced GFR, for example. To demonstrate this possible bias, we simulated an epidemiological study to assess this association (S3 File). We know that renal function declines with increasing age, an association that was evaluated in the MDRD study where GFR was measured directly as the renal clearance of ^125^I-iothalamate [1]. We then calculated the expected decline in renal function by age using both the exponential function [-2.03 ln (age)] derived from the MDRD study (measured slope) and the estimated GFR using the CKD-EPI formula (simulated slope). While the measured slope was 0.6 ml/min/1.73m^2^ per year, the simulated slope was 0.28 ml/min/1.73m^2^ per year, suggesting that the estimated GFR is underestimating the true decline in renal function (Fig 3). In this case, the lateral collinearity represents a clinically relevant issue, highlighting the need to use measured GFR.

Although not formally wrong, the interpretation of the correlation between age, gender, and ethnicity with the eGFR should be considered with caution because there is no easy mathematical computation model to assess the lateral collinearity.

## Supporting information

S1 File Code in R related to data and statistics. (R)

S2 File Sensitive analysis with simulated data for 1000 patients (age, ethnicity, gender, and creatinine) considering considered a reduction of serum creatinine with advanced age and in females and high creatinine values in black-ethnicity. In females, we considered the factor correction of 0.7 and in black -ethnicity the correction of 1.2. This correction factor was derived from MDRD. We also considered a reduction of creatinine with age in an exponential function (exp -0.2).
(HTML)

S3 File Sensitive analysis for a simulated epidemiologic study aims to demonstrate the collinearity in a practical example. The data show the relationship between eGFR by CKD-EPI with age though regression lines. The regression line was done by simulated data (simulated slope) and derived by the expected decline with age according to the MDRD study (measured slope).
(HTML)

## Abbreviations

BMI: body mass index
CKD-EPI: Chronic Kidney Disease Epidemiology Collaboration
eGFR: estimated glomerular filtration rate
FAS: full spectrum equation
GFR: Glomerular filtration rate
KDPI: Kidney Donor Profile Index
MDRD: Modification of Diet in Renal Disease Study Group
VIF: variance inflation factor
