# Supplementary material for: Recycling of predictors used to estimate glomerular filtration rate: Insight into lateral collinearity
Source: PLoS One. 2020 Feb 11;15(2):e0228842. doi: 10.1371/journal.pone.0228842 (PMC7012427; doi:10.1371/journal.pone.0228842)
Supplement: S2 File — In females, we considered the factor correction of 0.7 and in black -ethnicity the correction of 1.2. This correction factor was derived from MDRD. We also considered a reduction of creatinine with age in an exponential function (exp -0.2). (HTML) [file pone.0228842.s002.html]

eGFR


# eGFR

## Simulated sample considering the associations between creatinine and demographic factors

**Table 01. Simulated values and Estimated Glomerular Filtration rate in a simulated sample**

|  | Overall (N=1000) |
| --- | --- |
| **age** |  |
| Mean (SD) | 49.763 (7.631) |
| Range | 27.680 - 76.584 |
| **sex** |  |
| female | 492 (49.2%) |
| male | 508 (50.8%) |
| **creatinine** |  |
| Mean (SD) | 0.873 (0.334) |
| Range | 0.500 - 2.107 |
| **ethnicity** |  |
| non-Black | 569 (56.9%) |
| Black | 431 (43.1%) |
| **eGFR\_EPI** |  |
| Mean (SD) | 96.609 (25.090) |
| Range | 39.784 - 160.399 |
| **eGFR\_MDRD** |  |
| Mean (SD) | 101.477 (39.282) |
| Range | 36.841 - 226.712 |

**The relationship between the age, creatinine, sex and ethnic with estimated glomerular filtration rate (eGFR).**

```
## `geom_smooth()` using method = 'gam' and formula 'y ~ s(x, bs = "cs")'
```

## Linear model of age, sex and ethnicity with creatinine (outcome) and eGFR by CKD-EPI

```
model_3 <- lm(eGFR_EPI ~ age + sex + ethnicity, data = egf)
sjt.lm(model_3)
```

|  | eGFR\_EPI | | ||  |  | B | CI | p |
| (Intercept) |  | 117.78 | 107.49 – 128.07 | <.001 |
| age |  | -0.39 | -0.59 – -0.19 | <.001 |
| sex (male) |  | -6.40 | -9.46 – -3.33 | <.001 |
| ethnicity (Black) |  | 3.08 | -0.02 – 6.17 | .051 |
| Observations |  | 1000 | | |
| R2 / adj. R2 |  | .036 / .034 | | |
