# Supplementary material for: Recycling of predictors used to estimate glomerular filtration rate: Insight into lateral collinearity
Source: PLoS One. 2020 Feb 11;15(2):e0228842. doi: 10.1371/journal.pone.0228842 (PMC7012427; doi:10.1371/journal.pone.0228842)
Supplement: S3 File — The data show the relationship between eGFR by CKD-EPI with age though regression lines. The regression line was done by simulated data (simulated slope) and derived by the expected decline with age according to the MDRD study (measured slope). (HTML) [file pone.0228842.s003.html]

eGFR\_trail\_n


# eGFR\_trail\_n

# Simulated Epidemiological Study

Simulated sample considering the associations between creatinine and demographic factors

## Tree Plot

# eGFR CKD-EPI by Age

Relationship between eGFR by CKD-EPI with age in a simulated epidemiologic study. The expected decline in renal function was obtained from the MDRD study. In this study, the expected decline in renal function by age follows an exponential function. In the graph, the blue regression line represents the expected slope of renal function decline (-0.6 ml/min/1.73m2/year). The simulated slope is the red regression line (-0.28 ml/min/1.73m2/year) and represents the values obtained with the simulated data. The values obtained with the simulated data underestimating the true decline in renal function
